# Neural Network Cascade Optimizes MicroRNA Biomarker Selection for Nasopharyngeal Cancer Prognosis

**DOI:** 10.1371/journal.pone.0110537

**Published:** 2014-10-13

**Authors:** Wenliang Zhu, Xuan Kan

**Affiliations:** 1 Institute of Clinical Pharmacology, the Second Affiliated Hospital of Harbin Medical University, Harbin, China; 2 Department of Otolaryngology, the Second Affiliated Hospital of Harbin Medical University, Harbin, China; University of Torino, Italy

## Abstract

MicroRNAs (miRNAs) have been shown to be promising biomarkers in predicting cancer prognosis. However, inappropriate or poorly optimized processing and modeling of miRNA expression data can negatively affect prediction performance. Here, we propose a holistic solution for miRNA biomarker selection and prediction model building. This work introduces the use of a neural network cascade, a cascaded constitution of small artificial neural network units, for evaluating miRNA expression and patient outcome. A miRNA microarray dataset of nasopharyngeal carcinoma was retrieved from Gene Expression Omnibus to illustrate the methodology. Results indicated a nonlinear relationship between miRNA expression and patient death risk, implying that direct comparison of expression values is inappropriate. However, this method performs transformation of miRNA expression values into a miRNA score, which linearly measures death risk. Spearman correlation was calculated between miRNA scores and survival status for each miRNA. Finally, a nine-miRNA signature was optimized to predict death risk after nasopharyngeal carcinoma by establishing a neural network cascade consisting of 13 artificial neural network units. Area under the ROC was 0.951 for the internal validation set and had a prediction accuracy of 83% for the external validation set. In particular, the established neural network cascade was found to have strong immunity against noise interference that disturbs miRNA expression values. This study provides an efficient and easy-to-use method that aims to maximize clinical application of miRNAs in prognostic risk assessment of patients with cancer.

## Introduction

MicroRNAs (miRNAs) belong to a class of small (∼22 nt) endogenous non-coding RNA molecules. MiRNAs play vital roles in regulating mRNA expression and fine-tuning protein levels posttranscriptionally [1], [2]. Substantial evidence has shown that miRNAs may serve as promising therapeutic targets for clinical cancer treatment in the near future [3]–[5]. Meanwhile, the potential clinical applications of diagnostic and prognostic biomarkers are also widely studied and strongly suggest the utility of measuring circulating and biopsy tissue miRNAs [6]–[8]. Due to continual technological innovations in the past years, high-throughput methods such as miRNA microarray have been successful in the identification of potential biomarkers from thousands of mature miRNAs in humans [9], [10]. As a result, such efforts have led to an increasing accumulation of miRNA expression data in the public Gene Expression Omnibus (GEO) database [11].

Simultaneous detection of many miRNAs generates a huge dataset of biological data that requires significant computational analysis. Although the current miRNA detection technologies are already very well established, there is still no widely recognized method for analyzing the massive amount of data obtained by high-throughput methods [12]. The vast majority of previous studies assumed a linear relationship between miRNA expression and disease phenotype [13]–[15]. This led to wide application of straightforward statistical methods such as Student’s *t*-test or the analysis of variance test for between-group comparison of miRNA expression values. However, this assumption has not been specifically tested, or shown to be valid. Alternatively, rather than a linear relationship, we speculated that a nonlinear association may be possible between miRNA expression and disease phenotype. This assumption is primarily based on the knowledge that miRNAs play multi-faceted and complex roles in many biological processes [16]. If the nonlinear relationship is valid, it may imply that traditional miRNA expression data processing, analyzing, and modeling with linear methods are insufficient.

The improper selection of statistical or modeling methods may harm the potential performance of miRNAs as biomarkers and result in poor discrimination of patients [17], [18]. We propose one feasible way to address this issue through transforming miRNA expression values into a linear variable before establishing a diagnostic or prognostic model. Using this proposed method, the present study aims to provide a holistic and generic solution for miRNA biomarker selection and prediction model construction. In recent years, artificial neural network (ANN) modeling has been successfully applied in cancer diagnosis and management [19]–[21]. Herein, a novel artificial neural network (ANN) modeling method was established for this purpose: the neural network cascade (NNC), an extensible and pyramid-like cascade of small ANN units. Each small ANN unit has simple network architecture and is limited to dealing with only one task, such as data transformation, data integration, or prediction output. In theory, an NNC model can simultaneously accommodate and process large amounts of information in parallel. Even if a single input has poor predictive performance, as long as sufficient input information is given, an accurate final prediction is guaranteed. The number of input parameters included in the model depends on the accuracy requirements placed on the final prediction.

To better illustrate our method, we developed an NNC prognostic model for death risk assessment in patients with nasopharyngeal carcinoma (NPC) using a miRNA expression dataset retrieved from GEO (dataset ID: GSE32960). Our results suggest a nonlinear association between miRNA expression and the death risk of patients diagnosed with NPC. The established NNC model showed good prediction performance by accurately identifying high-risk patients, even in the case where miRNA expression levels were artificially disturbed. In summary, such an effort aims to analytically enhance the utility of miRNAs as clinical biomarkers for achieving accurate diagnosis and individualized cancer treatment. Our successful case study analysis of NPC prognosis using the novel NNC model suggests that this model will also be applicable to diagnosis and prognosis of other human diseases.

## Materials and Methods

### miRNA expression data: acquisition and pre-processing

The miRNA expression dataset for patients with NPC (GSE32960) was retrieved from GEO. Only the 312 NPC samples were included in our study. We downloaded the preprocessed microarray expression values for 873 miRNAs for each sample and recorded the survival status (alive: 0 or dead: 1) of the corresponding patient. The original microarray expression values of each miRNA were then normalized as numbers between 0 and 1 as calculated below:
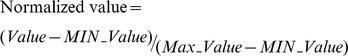



Max_Value and Min_Value are the maximum and minimum original miRNA expression values in the whole collection of samples, respectively. After that, the samples were randomly divided into two sets: a model training set (n = 208) and an external validation set (n = 104). For samples in the training set, the ANN software STATISTICA Neural Networks (SNN, Release 4.0E) was used to build ANN units, which transform miRNA expression values into miRNA scores for each of the 873 miRNAs. The ANN units have three layers: the input variable, output variable, and a function to connect the two. We used the imported normalized miRNA expression values as the input variable and survival status as the output variable. For the middle layer, the advanced version of Intelligent Problem Solver (IPS) tool was applied to build a radial basis function (RBF)-ANN with 11 hidden units. Network output values were referred to as miRNA scores, which were thought to be linearly associated with the death risk of patients. The nonparametric Spearman correlation coefficient (Spearman R) was calculated to assess the linear relationship between the normalized miRNA score and survival status for each patient.

### miRNA biomarker selection and ANN model building

Putative miRNAs biomarkers were ranked and selected on the basis of Spearman R values. In this study, we chose to retain only the nine miRNAs with the highest R values and discard the others. The normalized miRNA expression values and normalized miRNA scores of three miRNAs with the best Spearman R values (miR-29c, miR-34c-5p, and miR-93) were used to build the untransformed neural network models (UNN) and transformed neural network (TNN), respectively. Both models had the same network architecture (3-11-1). All of the miRNA scores of the nine miRNAs were then used for building the novel ANN model, which we named the neural network cascade (NNC). An NNC is composed of many ANN units. Each ANN unit is an independent ANN model. In an NNC model, the primary nine ANN units were used for the selected nine miRNAs to transform them from miRNA expression levels into miRNA scores. Each unit had a 1-11-1 network architecture. After that, a secondary ANN unit with a 3-11-1 framework was then built to integrate the outputs of the three data transformation units. A total of three such secondary units were needed for the nine miRNAs. Finally, a tertiary ANN unit was built to combine the outputs of the above three secondary ANN units. The ultimate output is a numerical prediction of the death risk of patients with NPC based on their miRNA gene expression signatures. Notably, we named all model outputs as miRNA scores, regardless of their origin from the ANN units or the composite models. Additionally, a detailed description of NNC model building was provided in Text S1.

### Internal and external validation

The holdout cross-validation method was used to conduct internal validation for each ANN unit by using the default settings of the IPS tool. The 208 training model samples were randomly divided into three sets, including training set, verification set, and testing set in a ratio of 2∶1∶1. Linear regression was used to assess the consistency of the training and testing set outputs. Similar correlation coefficients for the training and testing sets implies the given ANN unit has good generalization ability and *vice versa*. Furthermore, an independent set that consisted of 104 samples was used to perform external validation of the prediction accuracies of the NNC model. In addition to linear regression, a receiver operating characteristic (ROC) curve analysis was also performed to assess the prediction effects of the UNN, TNN, and NNC models by using the software MedCalc (version 13.0). The positive predictive value (PV) at each miRNA score criterion was calculated and used to estimate the probability of poor prognosis for the 104 patients in the external validation set.

### Statistical analysis

Student’s *t*-test was used for comparisons between two survival status groups of patients with NPC from various aspects, including miRNA expression, miRNA score, and probability of poor prognosis. Analysis of the area under the ROC curve (AUROC) was used to compared each risk prediction performance by miRNA scores of different miRNAs, miRNA expression, and scores of the same miRNA, or final outputs of different ANN models [22]. Differences were considered as statistically significant when *p*<0.05 for all the statistical methods used in this study.

## Results

### Nine miRNAs were selected as NPC prognostic biomarkers from the 873 measured miRNAs

**Figure 1:**
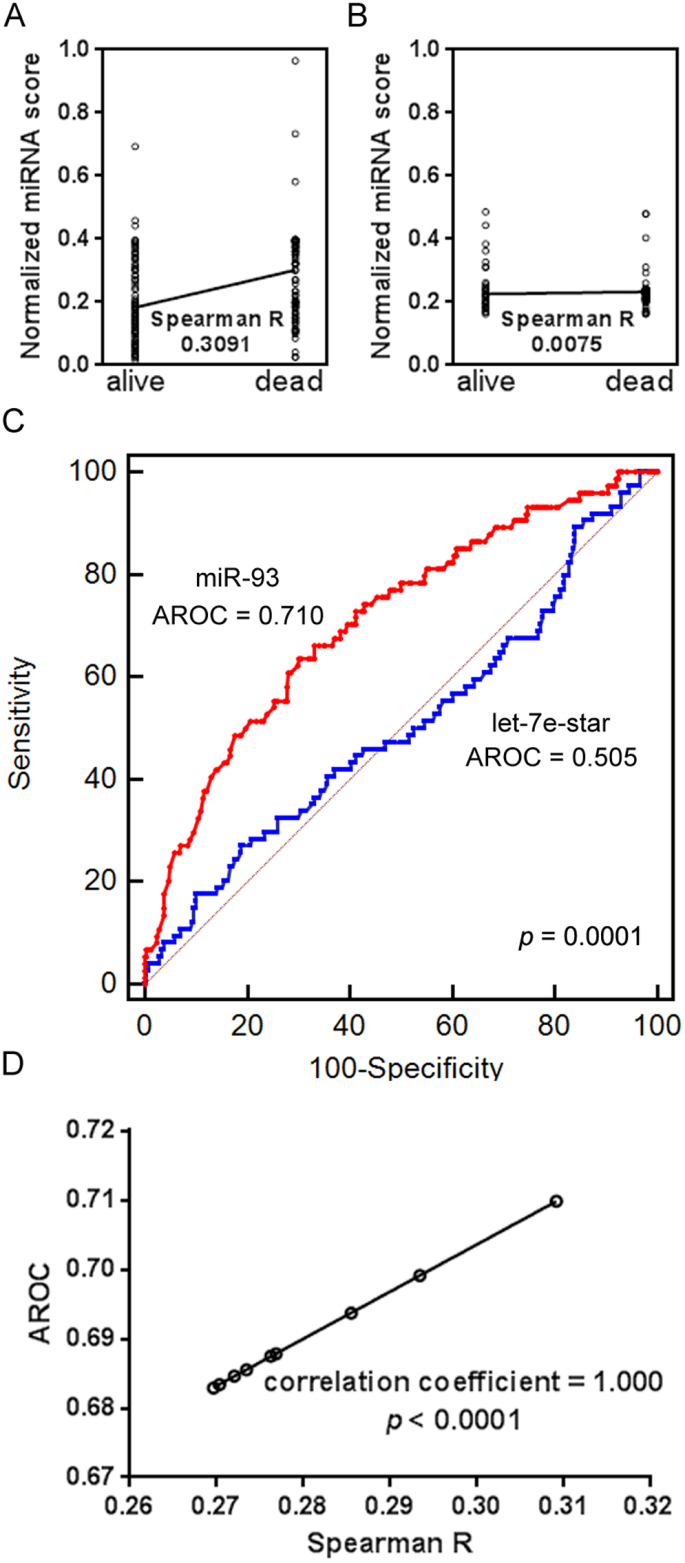
miRNA biomarker selection results. **A)** A significant linear relationship exists between the normalized miR-93 scores and patient survival status. Spearman R = 0.3091; *p*<0.0001. **B)** No significant linear relationship was found between normalized let-7e-star scores and patient survival status. Spearman R = 0.0075; *p*<0.895. **C)** AUROC comparison between the death risk prediction models using miR-93 and let-7e-star scores. A significant difference was observed (*p* = 0.0001). **D)** A perfect linear correlation relationship was found between Spearman R values and AUROCs (n = 9). *p*<0.0001.

First, we normalized and processed the original miRNA expression values that were downloaded from the GEO dataset of gene expression in patients with NPC (GSE32960). Next, the 312 patient samples were randomly divided into a model training set and an external validation set at a ratio of 2∶1. In the model training set, small ANN models with network architecture of 1-11-1 was applied to convert miRNA expression values into miRNA scores for each miRNA analyzed. The software GraphPad Prism 6.0 was then used to calculate the Spearman R between miRNA scores and patient survival status for each of the 873 miRNAs. Finally, among the 873 miRNAs, nine miRNAs with the highest Spearman R values were highlighted: miR-93, miR-29c, miR-34c-5p, miR-202, miR-145-star, miR-1292, miR-26a, miR-30e, and miR-15b (in descending order of Spearman R value). The miR-93 miRNA score showed the best linear correlation with survival status (Figure 1A, Spearman R = 0.3091). Comparably, the let-7-star miRNA score was found to be unrelated with NPC patient survival (Figure 1B, Spearman R = 0.0075). This result was further confirmed by our ROC analysis (Figure 1C). The AUROC of the prediction model using the miR-93 miRNA score was significantly higher than that of the prediction model using the miRNA score of let-7e-star (*p* = 0.0001). Furthermore, we calculated AUROCs for the other eight miRNAs that were selected as potential biomarkers for NPC prognosis. A stringent correlation relationship was revealed between the values of Spearman R and those of AUROCs (Figure 1D). This result suggests calculating Spearman R or AUROC leads to similar effectiveness in the ability to detect preferred biomarkers from miRNA microarray experiments.

### Expression of nine candidate miRNAs biomarkers was nonlinearly related with survival status

**Figure 2:**
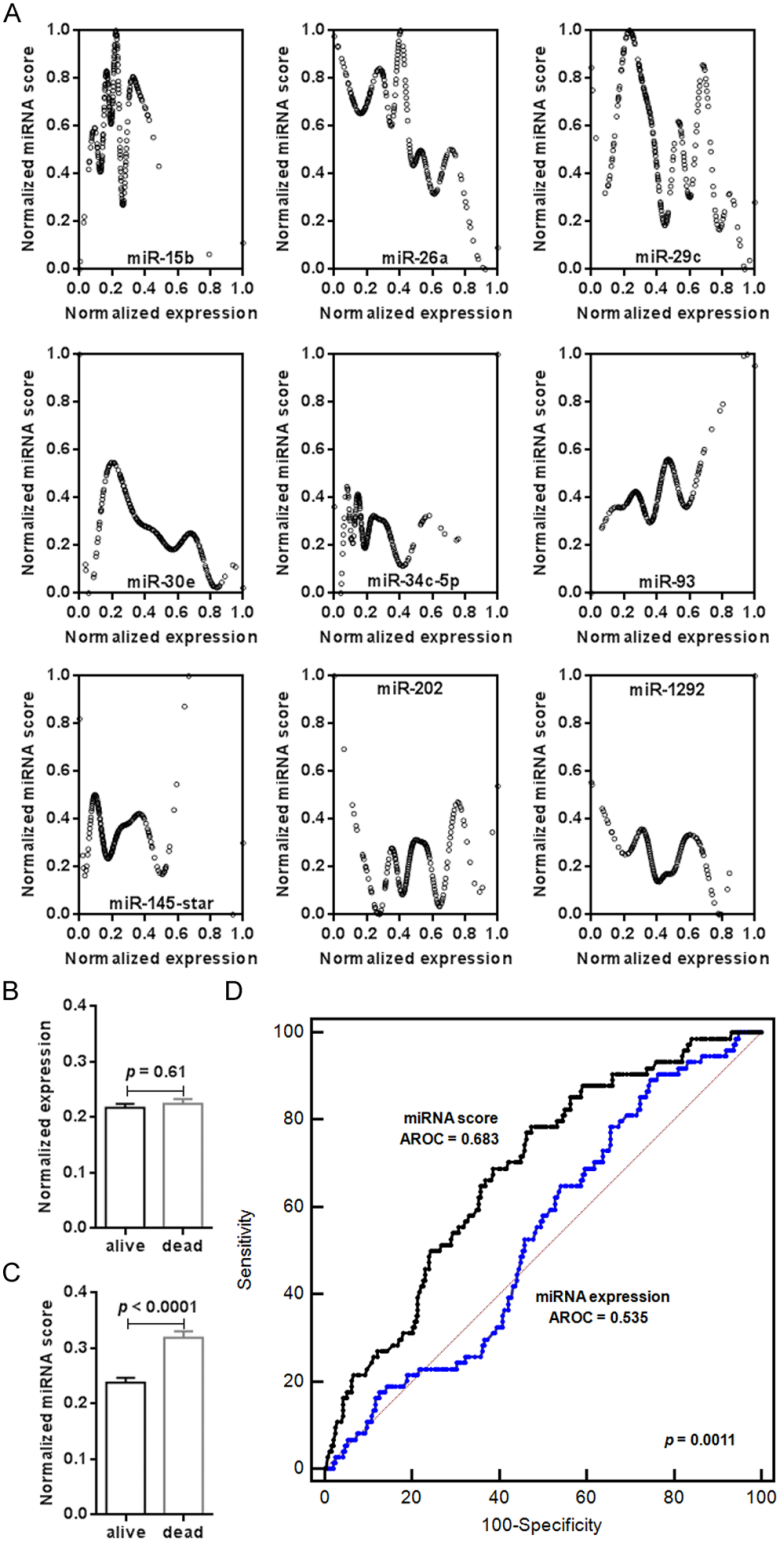
miRNA expression is non-linearly related with NPC patient death risk. **A)** Illustration of the relationship between normalized miRNA expression and normalized miRNA scores of the selected nine miRNA biomarkers. **B)** No significant difference was observed in normalized miR-15b expression between patients with survival statuses of ‘alive’ and ‘dead’. Mean ± SEM; *p* = 0.61. **C)** The miRNA scores of miR-15b were significantly different when patients with survival statuses of ‘alive’ and ‘dead’ were compared. Mean ± SEM; *p*<0.0001. **D)** AUROC comparison between the death risk prediction models using miRNA expression and miRNA scores of miR-15b, respectively. A significant difference was found (*p* = 0.0011).

Scatter plots were drawn to illustrate the relationship between miRNA expression and miRNA scores (Figure 2A). As a result, no linear relationship was detected between miRNA expression and miRNA scores for the nine selected candidate miRNA biomarkers. As the miRNA score is a linear variable assessing the death risk of patients with NPC, such a result indicates a nonlinear relationship between miRNA expression and patient survival statuses. This finding also implies that direct between-patient comparison of miRNA expression may not be suitable for predicting prognosis. The miRNA miR-15b was used to further examine this point. According to the Spearman R value, miR-15b was selected as one of the nine preferred miRNA biomarkers indicating NPC prognosis. However, we did not find any difference in miR-15b expression between the two patient groups with different survival statuses by Student’s *t*-test (Figure 2B). In contrast, our method of transforming miRNA gene expression values into the miRNA score enabled us to successfully distinguish between the two patient groups (Figure 2C). Compared with miRNA expression, the miRNA score gave a positive prediction, which was further verified by the ROC analysis (Figure 2D). Similar results were also observed in miR-34c-5p, miR-145-star, miR-202, and miR-1292 (Figure S1).

### The NNC model showed the best prediction of patient death risk

**Figure 3:**
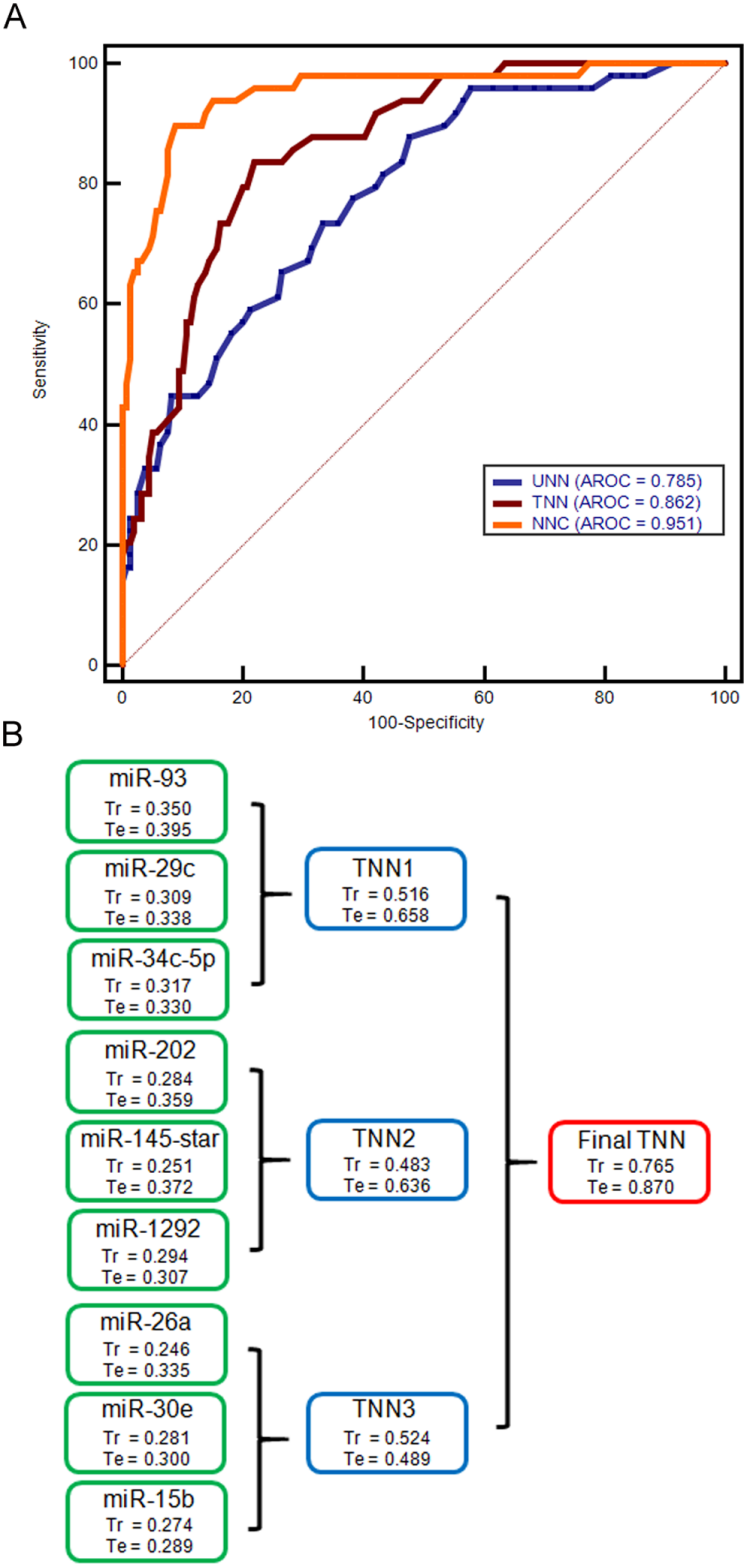
AUROC comparison of three ANN models (A) and results of internal validation of the NNC prediction model (B). UNN: untransformed neural network; TNN: transformed neural network; NNC: neural network cascade. Tr and Te represent correlation coefficients between the output variable and miRNA score of training set and testing set in each ANN unit, respectively.

In this study, we built three ANN models to further demonstrate the significance of linear transformation of miRNA expression values into a miRNA score. The UNN model was a traditional ANN model with a 3-11-1 network framework constructed using the normalized miRNA expression values of miR-29c, miR-34c-5p, andmiR-93 as input variables. With the same network framework, the TNN model used the normalized miRNA scores of these three miRNAs as input variables. ROC analysis reveals a better predictive performance of the TNN model than that of the UNN model (Figure 3A). The last ANN model we built was an NNC model, which had the most complex network framework, incorporating 13 ANN units as shown in Figure 3B. The NNC model has an AUROC of 0.951, which indicates this model has the best predictive ability to distinguish patients with different survival statuses (Figure 3A). Internal validation indicates that it has good generalization ability for prognosis prediction of patients beyond the modeling training set (Figure 3B).

### The NNC model showed strong immunity against disturbed miRNA expression

**Figure 4:**
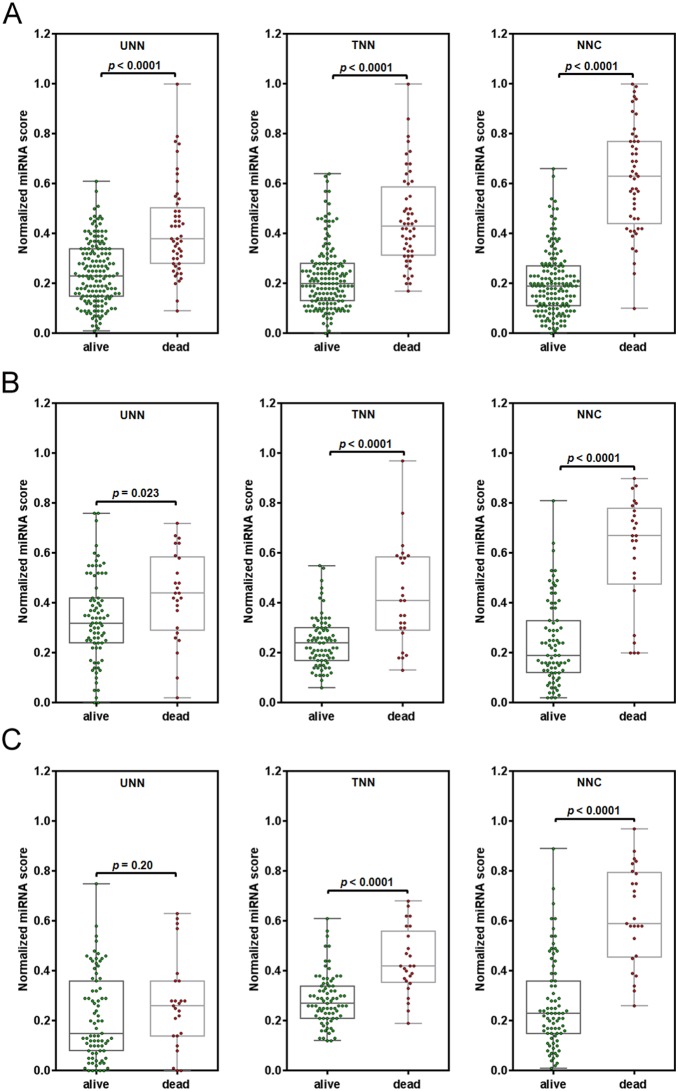
Scatter plots of miRNA scores exported from different ANN models (normalized). Comparisons of miRNA scores were performed between patients with different survival statuses in the model training set (**A**) and the external validation sets with normal miR-93 expression input (**B**) and with disturbed miR-93 expression input (**C**). UNN: untransformed neural network; TNN: transformed neural network; NNC: neural network cascade.

Scatter plots more clearly display the discriminative effect of different ANN models (Figure 4A). Compared with UNN or TNN, it is easy to identify that NNC had the best performance, despite the fact that all three models could significantly distinguish patients with the survival status of “dead” from those with the “alive” status (*p*<0.0001). The high predictive performance of NNC was confirmed when tested on the 104 patients used for external validation (Figure 4B). Further ROC analysis showed that the prediction accuracy was 83% for identifying high-risk patients by using the NNC model established here. Considering the diversity of the actual patients in the clinic, we also investigated the anti-interference capability of different models by replacing the miR-93 miRNA expression values with those of let-7e-star. In this study, the let-7e-star miRNA score had shown no relationship to the death risk of patients diagnosed NPC (Figure 1B and C). The result of this swap found that the UNN could not survive if the miR-93 expression values were seriously disturbed (Figure 4C). There is no significant difference in miRNA scores between two patient groups in this model (*p* = 0.20). Comparably, the other two models, especially the NNC model, still showed robust performance in distinguishing patients’ status.

**Figure 5:**
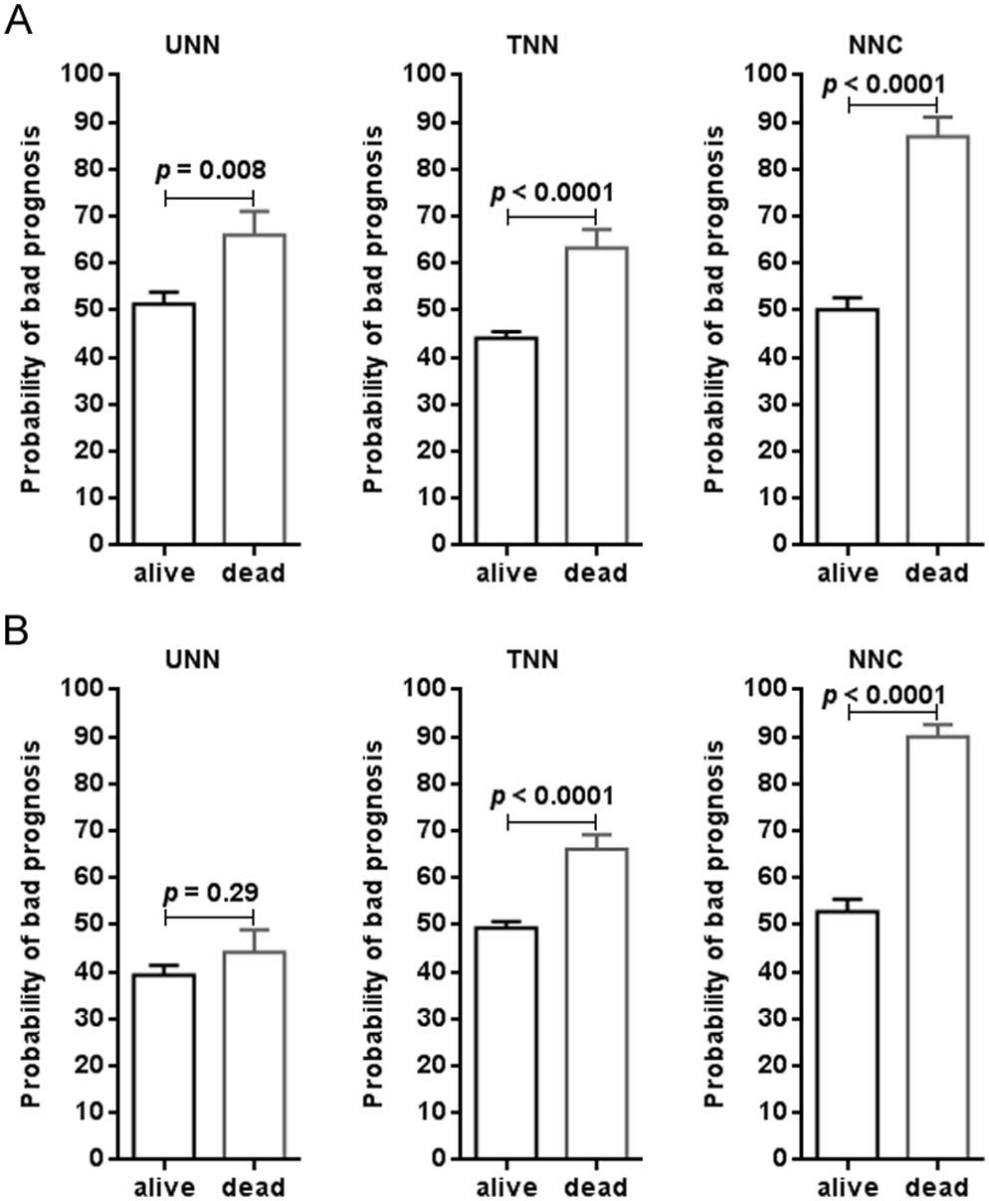
Comparison of bad prognosis likelihood between external validation patients with different survival statuses. **A)** Normal miR-93 expression input. **B)** Disturbed miR-93 expression input. UNN: untransformed neural network; TNN: transformed neural network; NNC: neural network cascade. All data are expressed as mean ± SEM.

Additionally, we evaluated the probability of a bad prognosisfor each patient with NPC. The mean probability of the patients with the survival status of ‘alive’ was 0.50, indicating that the death risk still exists for this group of patients (Figure 5A). Compared with UNN or TNN, NNC most accurately estimated the death risk of patients with the survival status of ‘dead’, even in the situation where the expression of miR-93 was seriously disturbed (Figure 5B). This finding suggests that the NNC model may have strong immunity against noise interference caused by unknown factors.

## Discussion

MiRNAs are widely thought to be the most promising class of endogenous substances for clinical diagnostic and prognostic biomarkers for cancer [23]. This conviction has prompted researchers worldwide to perform disease-specific miRNA expression profiling in an extensive field of cancer research [24], [25]. In this study, we attempt for the first time to present a generic method for translating miRNA expression data into clinically relevant language, such as the possibility of having cancer or the risk of bad prognosis due to suffering from cancer. Briefly, a computational model was constructed by integrating many small single-function ANN units into a cascaded network system. We named it the neural network cascade. We demonstrated that the neural network cascade was efficient for identifying the death risk of patients diagnosed with NPC.

The theoretical cornerstone for the NNC model established here is the assumption that miRNA expression may not be linearly associated with clinical phenotype indicators. This hypothesis is reasonable and realistic given the complexity of miRNAs involvement in human biology [16], [26], [27]. Based on this assumption, miRNA expression should be transformed into a linear variable before using it to evaluate the possibility of clinical consequences, such as that whether a patient is at high risk of death due to cancer. Our results support the validity of the hypothesis. We found a nonlinear relationship between miRNA expression and the death risk of patients with NPC. This finding implies the importance of miRNA expression data preprocessing before any miRNA-based clinical decisions are made.

Distinct from traditional artificial neural networks previously used in cancer diagnosis and management [19]–[21], the NNC did not directly use miRNA expression. Rather, the NNC first transforms miRNA gene expression into an miRNA score, a linear variable for assessing clinical phenotype. As a result, the miRNA score instead of miRNA expression was used for the purpose of selecting potential miRNA biomarkers and final decision-making. In the NNC model, the transformation and integration of data and final prediction output was achieved stepwise. This ensures overall computational simplification of the model’s operation. Another advantage of the NNC is that every miRNA is assigned an independent channel for information input. By such a design, if more miRNAs are needed for better prediction, one may expand the scale of the NNC model without increasing the network complexity of a single unit. This makes the NNC model freely expandable according to specific requirements. Expression data of different miRNAs can be considered as diverse information contributing to our existing knowledge of the death risk of patients. In our study, inclusion of more miRNAs was resulted in better predictions. The TNN contained three miRNAs and had an AUROC of 0.862. In contrast, the NNC model had an AUROC of 0.951, which contained 9 miRNAs. However, it is also possible that a larger NNC model for NPC prognosis could contain more than nine miRNAs. The nine miRNAs used in the NNC model here simply served as a methodology illustration.

Our external validation results of UNN and TNN indicate that linear transformation of miRNA expression notably improves the prediction effect of the model. Importantly, this procedure did not increase the number of miRNA biomarkers required, implying the advantage of using a cascaded structure of ANNs. Additionally, we found that the cascaded ANN constitution had a more robust performance than the traditional ANN model, where unexplained variability in miR-93 expression caused one ANN unit malfunction. Although unable to estimate the degree of such interference on disease prognosis in actual clinical settings, it remains possible that this variability will be an important factor that hinders miRNA-based prediction models in practice. Comparison of the TNN and NNC models suggests that inclusion of more miRNAs would increase robustness of the established ANN model against noise disturbance.

In conclusion, our study provided a rational and feasible method for miRNA biomarker selection and prediction model establishment. The advantage of a cascaded construction of small artificial neural network units is reflected from several aspects, including scalable capacity and flexible combination of miRNA expression inputs, better prediction with robust stability, and greater opportunities for meaningful modeling if the number of miRNA biomarkers is unrestricted. In the future, more attempts should be made to further validate the application of our approach by translating miRNA expression data into clinically relevant information for the diagnosis and prognosis of cancer.

## Supporting Information

Figure S1 **Comparison of miRNA expression and miRNA scores between the two patient groups with different survival statuses. A)** miR-26a; **B)** miR-29b; **C)** miR-30e; **D)** miR-34c-5p; **E)** miR-93; **F)** miR-145-star; **G)** miR-202; **H)** miR-1292. All data are expressed as mean ± SEM.
(TIF)

Text S1 **A step-to-step procedure for NNC model building.**
(DOCX)
