# Supplementary figures and images for: Neural Network Cascade Optimizes MicroRNA Biomarker Selection for Nasopharyngeal Cancer Prognosis
Source: PLoS One. 2014 Oct 13;9(10):e110537. doi: 10.1371/journal.pone.0110537 (PMC4195731; doi:10.1371/journal.pone.0110537)

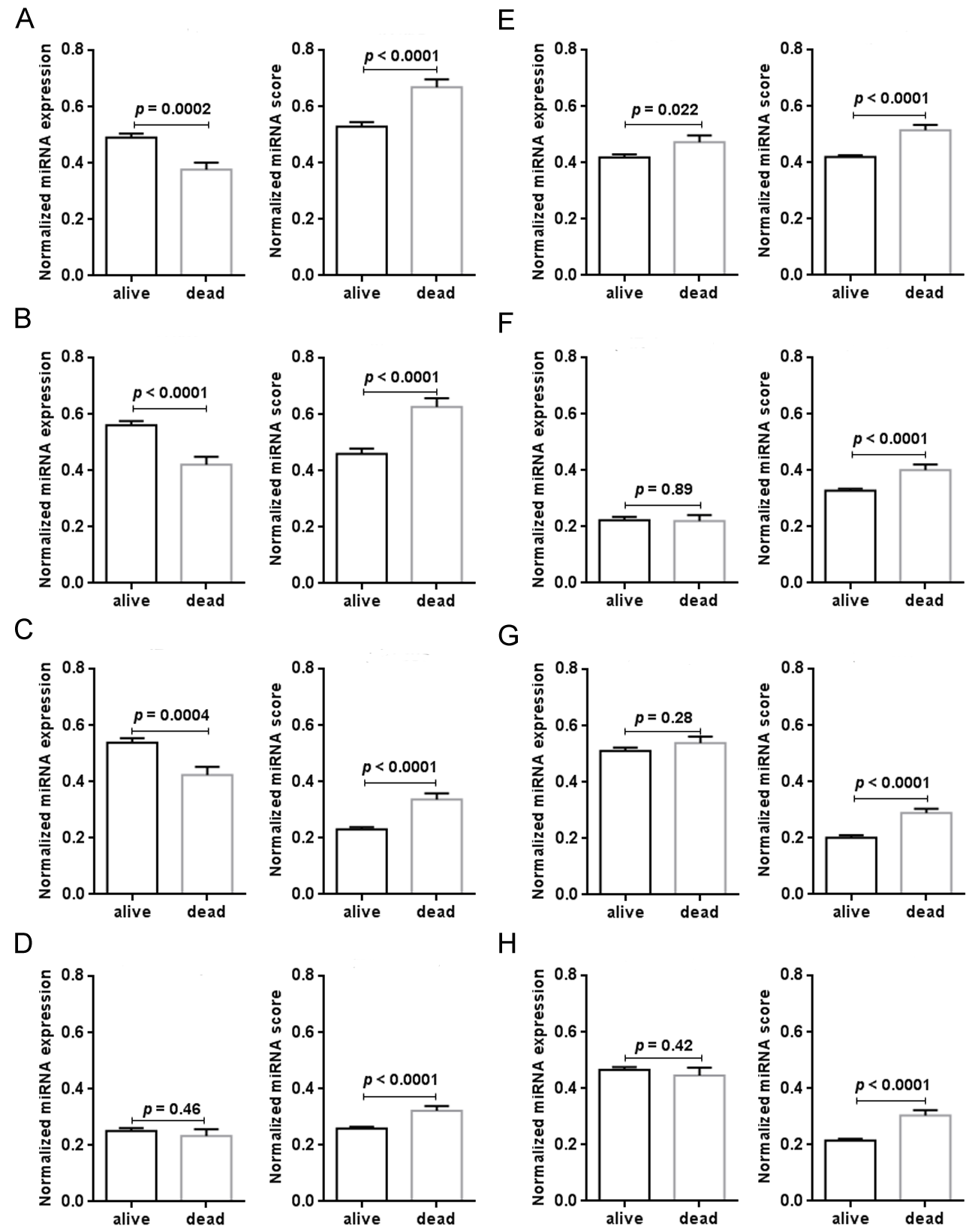

Supplement: Figure S1 — Comparison of miRNA expression and miRNA scores between the two patient groups with different survival statuses. A) miR-26a; B) miR-29b; C) miR-30e; D) miR-34c-5p; E) miR-93; F) miR-145-star; G) miR-202; H) miR-1292. All data are expressed as mean ± SEM. (TIF) [file pone.0110537.s001.tif]
