# Supplementary material for: Neural Network Cascade Optimizes MicroRNA Biomarker Selection for Nasopharyngeal Cancer Prognosis
Source: PLoS One. 2014 Oct 13;9(10):e110537. doi: 10.1371/journal.pone.0110537 (PMC4195731; doi:10.1371/journal.pone.0110537)
Supplement: Text S1 — A step-to-step procedure for NNC model building. (DOCX) [file pone.0110537.s002.docx]

**A step-to-step procedure for neural network cascade (NNC) model building**

**Step 1:** After starting the software STATISTICA Neural Networks (SNN, Release 4.0E), a data set should be firstly created in the Data Set Editor by importing the normalized microarray expression values of one of the 9 miRNAs as input and the corresponding patient survival statuses (alive: 0, dead: 1) as output. Notably, the number of rows in the data set should be correspondingly expended by adding cases to ensure that it can exactly contains such information of all the patients in the model training set.

**Step 2:** After the dataset is successfully filled, the Intelligent Problem Solver (IPS) tool needs to be activated for searching and building an ANN unit for the given miRNA. The version "Advanced" and the problem type of "Standard" should be sequentially selected. Radial basis function (RBF) is then chosen as the only network type. The opinion of "determine network complexity automatically" should be removed. This makes the number of hidden units fixed at 11. Duration of design process of "Throughout" is adopted to allow the IPS tool to have sufficient time to discover RBF networks with good performance as much as possible. A strict limitation of "maximum number of network" as 1 means that only the network with the best performance can be retained. Finally, the IPS network search result is saved into a .sta file named as the official symbol of the miRNA. As the normalized microarray expression values of only one miRNA are considered as inputs, the ANN unit saved in the file has a 1-11-1 network architecture.

**Step 3:** As 'Data Set' is selected in the drop-down list box of 'Run', the miRNA scores will be calculated for all the patients in the model training set. After that, these initial values of the miRNA scores should be pasted into the Excel software for further data normalization (see **Materials and Methods**). Finally, the statistical software Graphpad Prism v6.0 is used to calculate the Spearman R between the normalized miRNA scores and patient survival statuses.

**Step 4:** The above 3 steps should be repeated independently for 9 times until 9 primary ANN units are built and the normalized miRNA scores of the 9 miRNAs are obtained for each patient in the model training set.

**Step 5:** For further data integration, the 9 miRNAs need to be grouped into 3 three-miRNAs combinations according to the descending order of the Spearman R values that are calculated by Graphpad Prism v6.0. Again, with the normalized miRNA scores of three miRNAs as input variables and the corresponding patient survival statuses as the only output variable, the software SNN is used to build three secondary ANN units with a 3-11-1 framework for the 3 three-miRNAs combinations, respectively. Briefly, the IPS tool is used for network searching and building as described in **Step 2**. Notably, no subset of the three input variables is considered. The IPS network search results are sequentially saved into three .sta files named as secondary1, secondary2 and secondary3, respectively.

**Step 6:** In each secondary ANN units, the miRNA scores will be calculated for all the patients in the model training set by selecting 'Data Set' to run the network calculation. After that, the initial values of the miRNA scores should be pasted into the Excel software for further data normalization. In the end, each patient in the model training set is assigned with three miRNA scores (normalized) that are obtained from the three secondary ANN units, respectively.

**Step 7:** For obtaining the ultimate NNC prediction, the software SNN is used to build a tertiary ANN unit with a 3-11-1 framework, in which the three miRNA scores (normalized) from the secondary ANN units are imported as inputs and the corresponding patient survival statuses is used as output. After that, the IPS tool is used for network searching and building as described in **Step 2**. No subset of the three input variables is considered. The IPS network search result is saved into a .sta file named as final. The initial values of the miRNA scores exported from the tertiary ANN unit should be then pasted into the Excel software for miRNA score normalization.
